# Economic Evaluation of First-Line Camrelizumab for Advanced Non-small-cell Lung Cancer in China

**DOI:** 10.3389/fpubh.2021.743558

**Published:** 2021-12-10

**Authors:** Guiyuan Xiang, Lingna Gu, Xuan Chen, Fan Wang, Bohua Chen, Jie Zhao, Yun Lu, Feng Chang, Yumei Zhu

**Affiliations:** ^1^School of International Pharmaceutical Business, China Pharmaceutical University, Nanjing, China; ^2^Department of Pharmacy, Affiliated Hospital of Nantong University, Nantong, China; ^3^Department of Pharmacy, The First Affiliated Hospital of Zhengzhou University, Zhengzhou, China

**Keywords:** non-small-cell lung cancer, cost-effectiveness analysis, camrelizumab, China, first-line treatment

## Abstract

**Background:** As the first domestic PD-1 antibody approved for lung cancer in China, camrelizumab has exhibited proven effectiveness for non-small-cell lung cancer (NSCLC) patients. However, the cost-effectiveness of this new regimen remains to be investigated.

**Objective:** To evaluate the cost-effectiveness of camrelizumab combination therapy vs. chemotherapy for previously untreated patients with advanced, non-squamous NSCLC without Alk or Egfr genomic aberrations from the perspective of China's healthcare system.

**Methods:** Based on the CameL trial, the study developed a three-health state Markov model to evaluate the cost-effectiveness of adding camrelizumab to chemotherapy compared to chemotherapy alone in NSCLC patients. The analysis models were conducted for patients unselected by PD-L1 tumor expression (the base case) and the patient subgroup with PD-L1-expressing tumors (≥1%). Primary model outcomes included the costs in US dollars and health outcomes in quality-adjusted life-years (QALYs) as well as the incremental cost-effectiveness ratio (ICER) under a willingness-to-pay threshold of $31,500 per QALY. Additionally, a scenario analysis that adjusted within-trial crossover was employed to evaluate camrelizumab combination therapy compared to chemotherapy without subsequent use of PD1/PD-L1 antibodies.

**Results:** Camrelizumab combination therapy was more costly and provided additional 0.11 QALYs over chemotherapy in the base case analysis (0.86 vs. 0.75 QALYs), 0.12 QALYs over chemotherapy in the subgroup analysis (0.99 vs. 0.88 QALYs), and 0.34 QALYs over chemotherapy in the scenario analysis (0.86 vs. 0.52 QALYs). Correspondingly, the ICER was $63,080 per QALY, $46,311 per QALY, and $30,591 per QALY, in the base case, the subgroup, and the scenario analysis, respectively. One-way sensitivity analyses revealed that ICERs of the base case and the subgroup analysis were most sensitive to the cost of camrelizumab, the cost of pemetrexed. Besides, the base case and subgroup analysis were more sensitive to the risk of neutrophil count decreased in the camrelizumab and the utility of stable disease, respectively.

**Conclusion:** Although camrelizumab combination therapy is not cost-effective as first-line therapy for NSCLC patients in China in the base case, adjusting within-trial crossover would move the treatment regimen toward cost-effectiveness in the scenario analysis.

## Introduction

Non-small-cell lung cancer (NSCLC) is the most common subtype of lung cancer, accounting for ~85% of lung cancer diagnoses and the leading cause of cancer-related mortality in China (1, 2). Platinum-based regimens represented the cornerstone of first-line treatment for NSCLC for almost a decade, while patients receiving this standard chemotherapy only have a 5-year survival rate of 15% (3). Although targeted therapies have redefined treatment options for patients with driver-mutated NSCLC [e.g., anaplastic lymphoma kinase (ALK)-rearranged, epidermal growth factor receptor (EGFR)-mutant NSCLC], these therapies are ineffective in patients whose tumors lack these genetic alterations (4). Since the United States (US) Food and Drug Administration granted nivolumab approval for patients with metastatic NSCLC in 2015, immune checkpoint inhibitors (ICIs) targeting programmed cell death 1 (PD-1)/programmed cell death ligand 1 (PD-L1) have become integrated into the treatment of such patients, leading to historical developments in the treatment of non-driver-mutated NSCLC (5).

Camrelizumab, a newly developed monoclonal antibody against PD-1, exhibits good clinical benefit and acceptable safety profile in multiple tumor types, including Hodgkin Lymphoma, hepatocellular carcinoma, and esophageal squamous cell carcinoma (6). Additionally, the CameL trial (ClinicalTrials.gov identifier NCT03134872), a multicentre randomized phase 3 trial, evaluated the combination of camrelizumab and pemetrexed-platinum chemotherapy (camrelizumab combination therapy) for previously untreated patients with advanced, non-squamous NSCLC without *Alk* or *Egfr* genomic aberrations. In this trial, camrelizumab significantly prolonged median progression-free survival (PFS) by 3 months [11.3 vs. 8.3 months, hazard ratio (HR), 0.60; 95% confidence interval (CI), 0.45–0.79] and the median overall survival (OS) by 7.4 months (27.9 vs. 20.5 months, HR, 0.73, IC, 0.55–0.96) for patients receiving camrelizumab in comparison to placebo (7, 8). Owing to the synergistic effect and improved efficacy, in 2020, the National Medical Products Administration (NMPA) approved the incorporation of camrelizumab to pemetrexed-platinum chemotherapy as first-line treatment for patients with advanced, non-squamous NSCLC without *Alk* or *Egfr* tumor alterations in China (9).

Although this treatment regimen exhibited proven effectiveness, the question of whether its cost is proportional to its clinical value was insufficiently considered. In low- and middle-income countries like China, due to a relatively low willingness-to-pay (WTP) threshold, most published economic evaluations demonstrated that innovative PD-1/PD-L1 antibodies, including pembrolizumab (10, 11), atezolizumab (12), nivolumab (13), etc., were not cost-effective at public list prices as first-line treatment for NSCLC and could impose a profound financial consequence on cancer treatment spending. However, camrelizumab may provide a treatment option with cost-effectiveness as its manufacturer cut the original price of camrelizumab roughly 85% off for inclusion in the newest National Reimbursement Drug List (NRDL) of China. Therefore, it is important to analyze the impact of price reduction on the economic value of this treatment regime for patients suffering from NSCLC.

The aim of the present study was to analyze the cost-effectiveness of camrelizumab combination therapy in the first-line setting for advanced, non-squamous patients without *Alk* or *Egfr* genomic tumor aberrations, practically from the perspective of healthcare systems in resource-limited countries such as China.

## Methods

### Model Overview

From **t**he perspective of China's healthcare system, this study used a 3-state Markov model to evaluate the cost and effectiveness associated with camrelizumab combination therapy as first-line treatment for advanced non-squamous NSCLC without *Alk* or *Egfr* alteration. On the basis of the CameL trial, patients without previous systemic chemotherapy entered the model at an average age of 60. Two treatment options were included: camrelizumab 200 mg once every 3 weeks in combination with 4–6 cycles of carboplatin [area under curve (AUC), 5 mg/mL per min] and pemetrexed (500 mg/m^2^) or carboplatin (AUC 5 mg/mL per min) and pemetrexed (500 mg/m^2^) every 3 weeks, followed by maintenance therapy with camrelizumab up to 2 years plus pemetrexed or pemetrexed alone until disease progression, unacceptable toxicity, death, consent withdrawal, investigator decision, or study completion. All simulated patients entered the model in PFS state and could move to progressive disease (PD) and death overtime. The model included only direct medical care costs. The primary model outcomes were the average cost of each treatment strategy expressed in 2021 US dollars, the average number of life years (LYs) and quality-adjusted life-years (QALYs) gained, and the incremental cost-effectiveness ratio (ICER). The model adopted a lifetime horizon for the base case. The length of the cycle was 3 weeks. Cost and health outcomes were discounted at an annual rate of 5%. According to the recommendation of the World Health Organization (WHO), a WTP threshold of $31,500 per QALY (triple GDP per capita in China) was used to determine cost-effectiveness.

All patients who began in PFS state received first-line treatment, camrelizumab combination therapy or chemotherapy alone, respectively. Given a substantive number of early treatment discontinuations in the CameL trial, the model adjusted downward the PFS curve by applying the ratio of median time on treatment to median PFS at each week (8). After progression, the patients were assumed to receive second-line therapy. In the base case, 58% of patients who progressed in camrelizumab combination therapy group and 70% of patients who progressed in chemotherapy group received subsequent therapy, according to the CameL trial. For the sake of simplicity, the model assumed that patients without subsequent therapy after progression only received supportive care. The Supplementary Material provided these regimens and probabilities of subsequent-line treatments.

### Model Survival and Progression Risk Estimates

The probabilities of death from both PFS and PD state for each treatment were estimated based on the overall survival Kaplan-Meier curves of the CameL trial. GetData Graph Digitizer (version 2.26, http://getdata-graph-digitizer.com/) was used to digitize survival data from published curves. Then the study used the algorithm derived by Hoyle et al. to generate pseudo-individual patient data (14). According to the Akaike information criterion and the Bayesian information criterion, the best-fitting parametric distributions were selected among the Weibull, exponential, log-logistic, and log-normal distributions. The same approach was employed for estimating probabilities from PFS to PD state.

Within-trial crossover was permitted in the CameL trial. In total, 79 patients (46%) who showed radiological disease progression could crossover from chemotherapy to camrelizumab monotherapy. In order to remove the effects of within-trial crossover, the conversion of chemotherapy group was adjusted by Rank-Preserving Structural Failure Time (RPSFT) Model when extrapolating the OS curve (8).

### Cost and Utility Estimates

Direct medical care costs include the costs of drugs, management of adverse events (AEs), routine follow-up, best supportive care, and palliative care (see Table 1). All costs in this study were reported in January 2021 US dollar with an exchange rate of US $1 = 6.4769 Chinese yuan. The prices of therapeutic drugs were derived from the average purchase price of Chinese hospitals in the price database. The model assumed patients had an average weight of 65 kg and a body surface area of 1.72 m^2^, with a serum creatinine concentration of 56.7 μmol/L. The grade 3–5 AEs with a frequency of >5% were included as important risks of drug treatment. Since the Camel trial could not provide AEs costs and health utilities, relevant data were derived from published literatures. More information about the details of costs and health utilities were provided in Table 1.

**Table 1:** Model inputs.

**Parameter**	**Base case**	**Range**		**Distribution**	**Source**
		**Low**	**High**		
**Treatment cost ($)**					
Camrelizumab per cycle	452.08	361.66	542.50	Gamma	Local market
Carboplatin per cycle	17.65	14.12	21.18	Gamma	Local market
Pemetrexed per cycle	1,103.30	882.64	1,323.96	Gamma	Local market
Docetaxel per cycle	94.10	75.28	112.92	Gamma	Local market
Gefitinib per cycle	161.47	129.18	193.76	Gamma	Local market
Bevacizumab per cycle	1,788.42	1,430.73	2,146.10	Gamma	Local market
Nivolumab per cycle	4,283.44	3,426.75	5,140.13	Gamma	Local market
Supportive care per cycle	338.00	270.40	405.60	Gamma	(15)
Routine follow-up per cycle^a^	85.71	68.57	102.85	Gamma	(15)
Palliative care per event	2,464.50	1,971.60	2,957.40	Gamma	(15)
**Cost of managing adverse events ($)**					
Neutrophil count decreased	175.37	140.30	210.44	Gamma	(16)
Anemia	101.02	80.82	121.22	Gamma	(17)
Platelet count decreased	603.79	483.03	724.55	Gamma	(18)
**Risk of adverse events in**					
**camrelizumab group (grade III–IV)**					
Neutrophil count decreased	0.38	0.34	0.42	Beta	(7)
Anemia	0.19	0.17	0.21	Beta	(7)
Platelet count decreased	0.17	0.15	0.19	Beta	(7)
**Risk of adverse events in**					
**chemotherapy group (grade III–IV)**					
Neutrophil count decreased	0.30	0.27	0.33	Beta	(7)
Anemia	0.11	0.10	0.12	Beta	(7)
Platelet count decreased	0.12	0.11	0.13	Beta	(7)
**Health utility**					
Stable disease	0.81	0.73	0.90	Beta	(19)
Disease progression	0.58	0.52	0.64	Beta	(20)
**Health disutility**					
Neutrophil count decreased	0.20	0.18	0.22	Beta	(21)
Anemia	0.07	0.07	0.08	Beta	(21)
Platelet count decreased	0.11	0.10	0.12	Beta	(22)
Discount rate	0.05	0.00	0.08	Fixed in PSA	—

*PSA, probabilistic sensitivity analysis*.

a *The cost of routine follow-up included the cost of outpatient physician visit, hospitalization, and laboratory tests*.

### Sensitivity and Scenario Analyses

One-way deterministic sensitivity analyses were conducted on key model variables to assess their impacts of uncertainty on cost-effectiveness results. The ±20% ranges were applied for costs, and ±10% for utilities and risks of AEs if 95% CI ranges were not available. Probabilistic sensitivity analyses (PSA) were performed to assess the robustness of results by 1,000 Monte Carlo simulations. The subgroup analysis was conducted to evaluate the cost-effectiveness of camrelizumab in patient population with PD-L1-expressing tumors (≥1%). The present study also employed a scenario analysis adjusting within-trial crossover to explore the value of camrelizumab, in view of substantial within-trial crossover from chemotherapy to camrelizumab monotherapy.

## Results

### Base-Case Results

In the base case analysis, pemetrexed-platinum chemotherapy treatment was associated with a mean cost of $12,983 and a mean quality-adjusted survival of 0.75 QALY in patients unselected by PD-L1 tumor expression. Adding camrelizumab to pemetrexed-platinum chemotherapy resulted in a mean cost of $19,921 and a mean quality-adjusted survival of 0.86 QALY, yielding an estimated ICER of $63,080 per QALY (Table 2). These results indicated that camrelizumab combination therapy was not cost-effective as first-line treatment for unselected patients with non-squamous, advanced NSCLC without *Alk* or *Egfr* genomic aberrations.

**Table 2:** Discounted incremental cost-effectiveness of camrelizumab.

				**Incremental**			**ICER (incremental cost/QALY, $)**
**Analysis**	**Total cost, $**	**LYs**	**QALYs**	**Cost, $**	**LYs**	**QALYs**	
**Base case**						
Camrelizumab	19,921	1.36	0.86	6,938	0.18	0.11	63,080
Chemotherapy	12,983	1.18	0.75	NA	NA	NA	NA
**Sensitivity analysis**						
Camrelizumab	20,631	1.54	0.99	5,738	0.18	0.12	46,311
Chemotherapy	14,894	1.36	0.88	NA	NA	NA	NA
**Scenario analysis**						
Camrelizumab	19,921	1.36	0.86	10,508	0.55	0.34	30,591
Chemotherapy	9,413	0.81	0.52	NA	NA	NA	NA

*LYs, life-years; QALYs, quality-adjusted life-years; ICER, incremental cost-effectiveness ratio; NA, not applicable*.

### Subgroup Analysis

For the patient subgroup with PD-L1-expressing tumors (≥1%), camrelizumab combination therapy was associated with an incremental cost of $5,738 and an incremental QALY of 0.12 vs. chemotherapy alone, with an ICER of $46,311 per QALY (Table 2). Although the cost-effectiveness of camrelizumab combination therapy remained poor in the subgroup with PD-L1 positive tumors, the ICER of PD-L1 positive patients was more favorable than that of unselected patients in the base case.

### Scenario Analysis

To minimize potential bias and assess the OS accurately, the present study used adjusted OS conducted by the RPSFT model from the recent update analysis of the CameL trial (8). In the scenario with crossover-adjusted OS, camrelizumab combination therapy had an incremental cost of $10,508 and an incremental QALY of 0.34, for an improved ICER of $30,591 per QALY compared to chemotherapy (Table 2). After the adjustment for within-trial crossover, from the perspective of China's healthcare system, camrelizumab combination therapy was cost-effective as first-line treatment for patients with advanced, non-squamous NSCLC without *Alk* or *Egfr* genomic aberrations.

### Sensitivity Analysis

**Figure 1:**
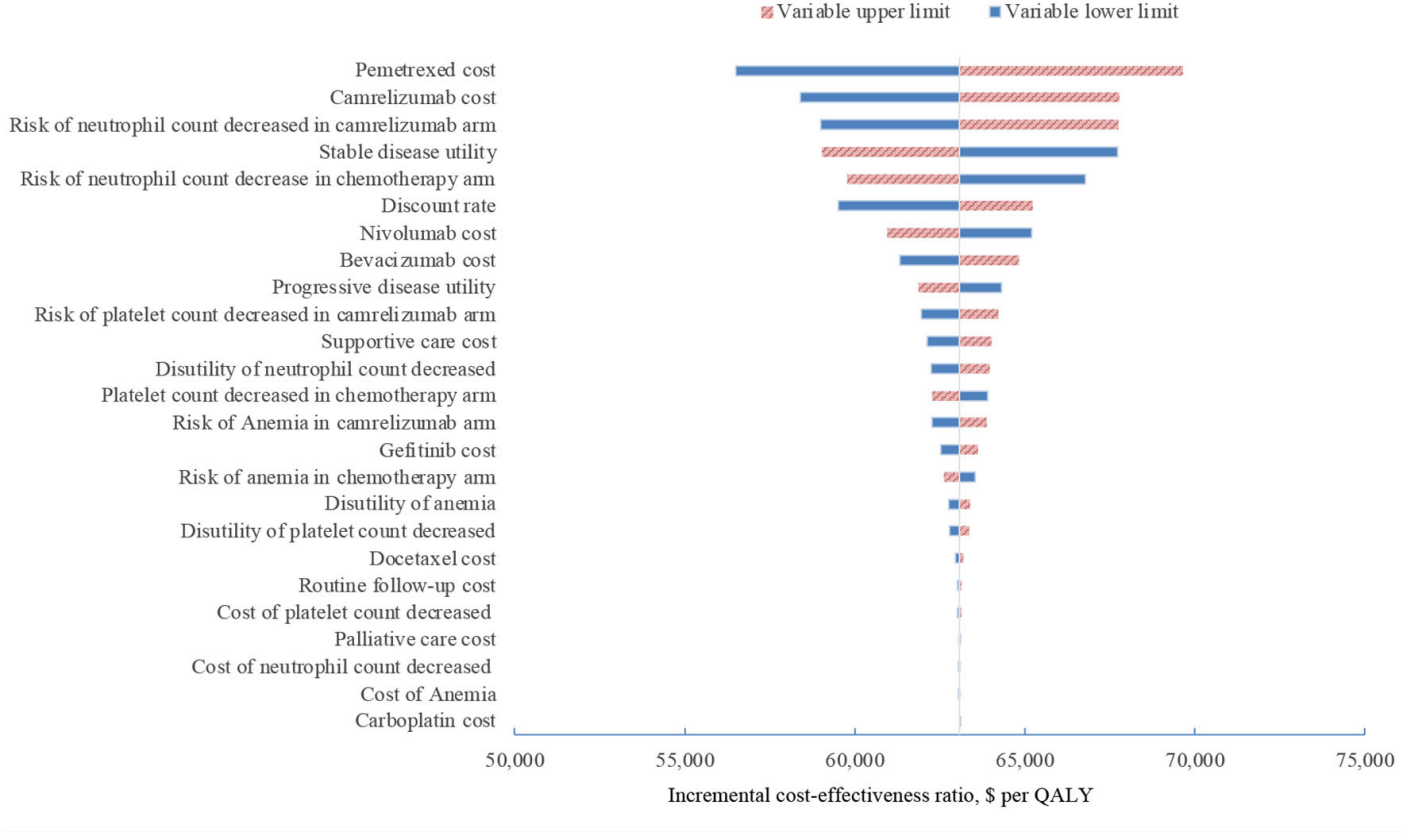
Deterministic sensitivity analysis for the base case analysis. QALY, quality-adjusted life-years.

**Figure 2:**
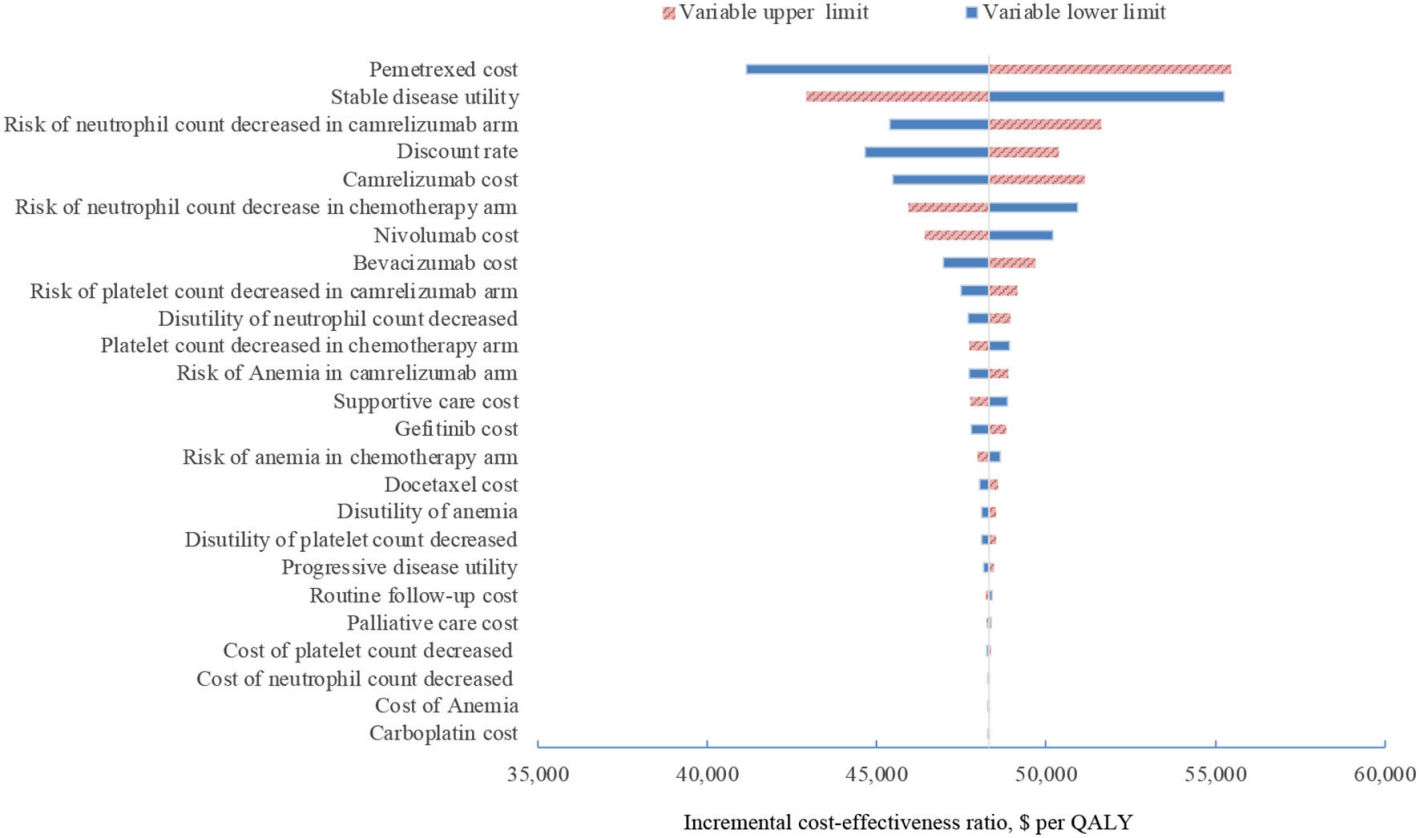
Deterministic sensitivity analysis for the subgroup analysis. QALY, quality-adjusted life-years.

**Figure 3:**
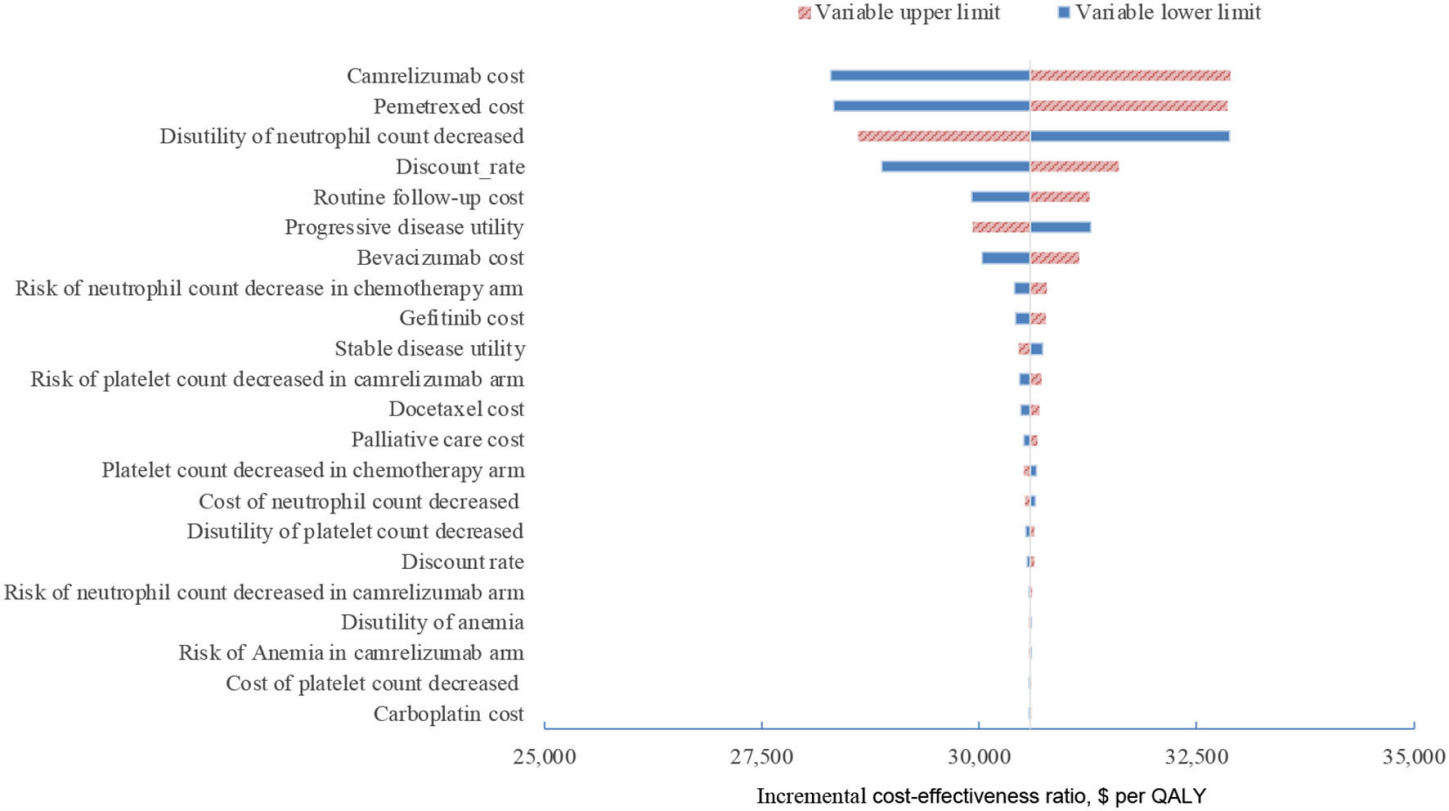
Deterministic sensitivity analysis for the scenario analysis. QALY, quality-adjusted life-years.

In one-way sensitivity analyses conducted for both unselected patients and the subgroup with PD-L1 positive tumors, camrelizumab combination therapy was not cost-effective at any of the tested variable upper or lower limits, assuming a WTP threshold of $ 31,500 per QALY (Figures 1, 2). However, the ICER of this treatment regimen decreased significantly at the lower limit of the pemetrexed cost ($56,518 per QALY in the base case and $41,146 per QALY in subgroup analysis) and the lower limit of the risk of neutrophil count decreased in the camrelizumab group ($58,996 per QALY in the base case and $45,378 per QALY in subgroup analysis). In addition, the ICER in the base case would decline to $58,385 per QALY when the cost of camrelizumab approached its lower limit. That in subgroup analysis would decrease to $42,927 per QALY at the upper limit of stable disease utility. In the crossover-adjusted scenario, camrelizumab combination therapy would not be cost-effective at the upper limit of the camrelizumab cost and pemetrexed cost and the lower limit of the disutility of neutrophil count decreased. In the crossover-adjusted scenario, camrelizumab combination therapy would not be cost-effective at the upper limit of the camrelizumab cost and pemetrexed cost and the lower limit of the disutility of neutrophil count decreased (Figure 3).

**Figure 4:**
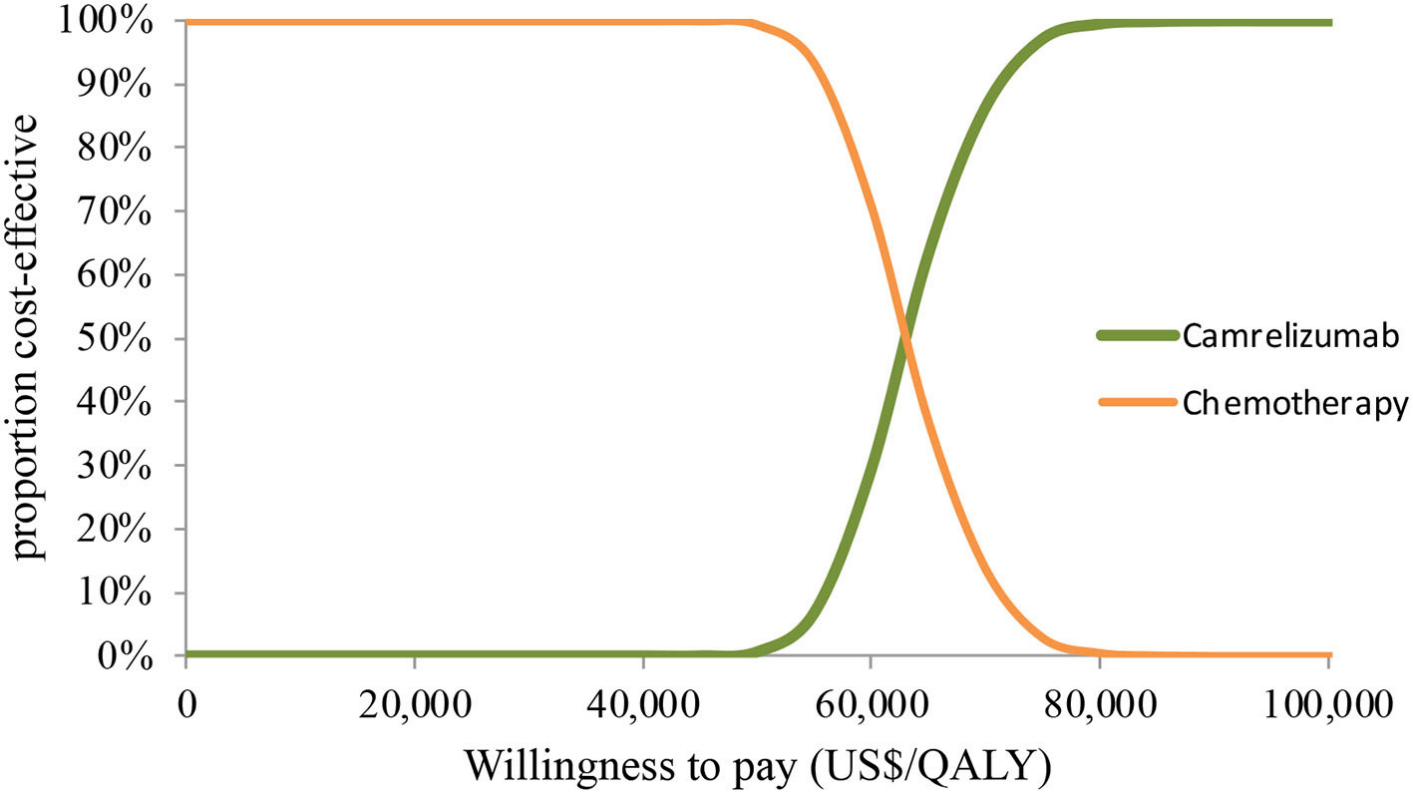
Cost-effectiveness acceptability curve for the base case analysis. QALY, quality-adjusted life-years.

**Figure 5:**
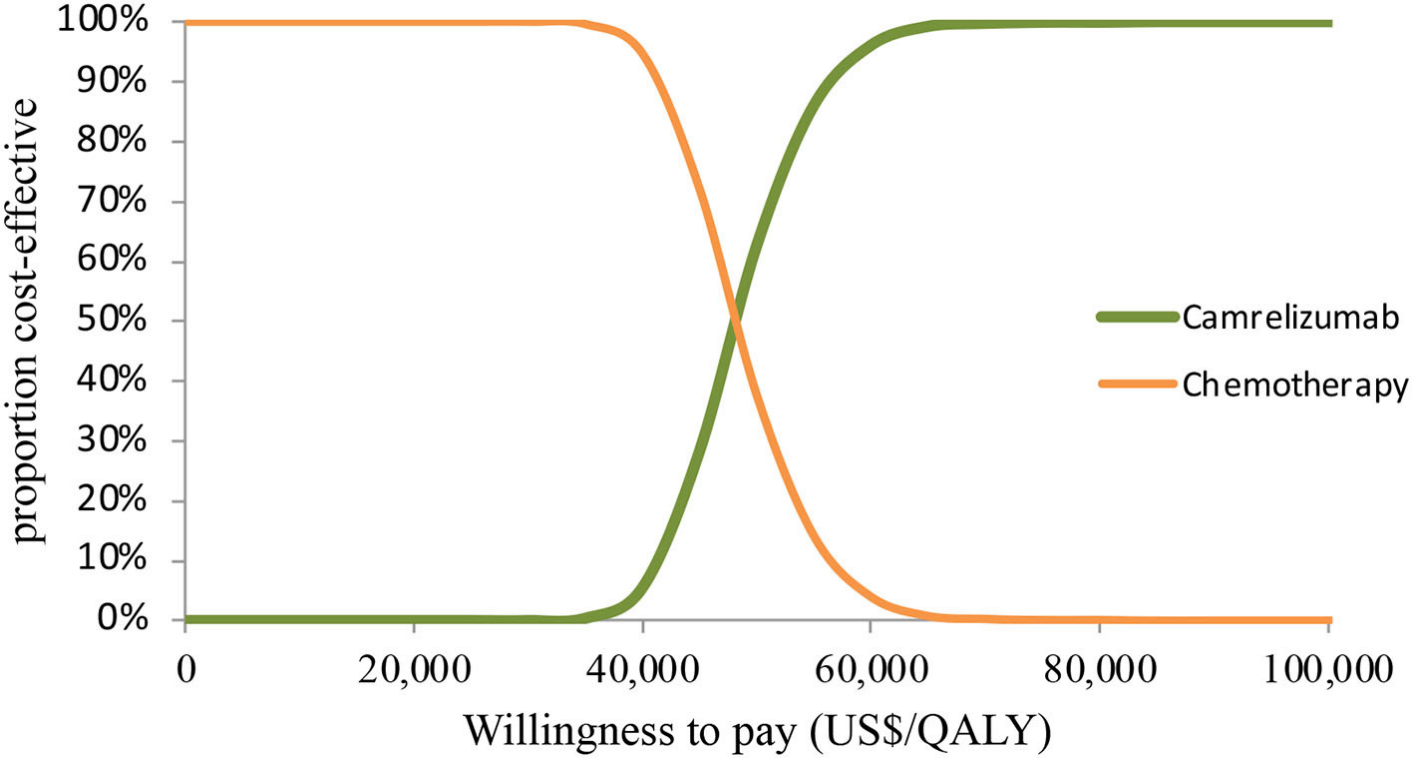
Cost-effectiveness acceptability curve for the subgroup analysis. QALY, quality-adjusted life-years.

**Figure 6:**
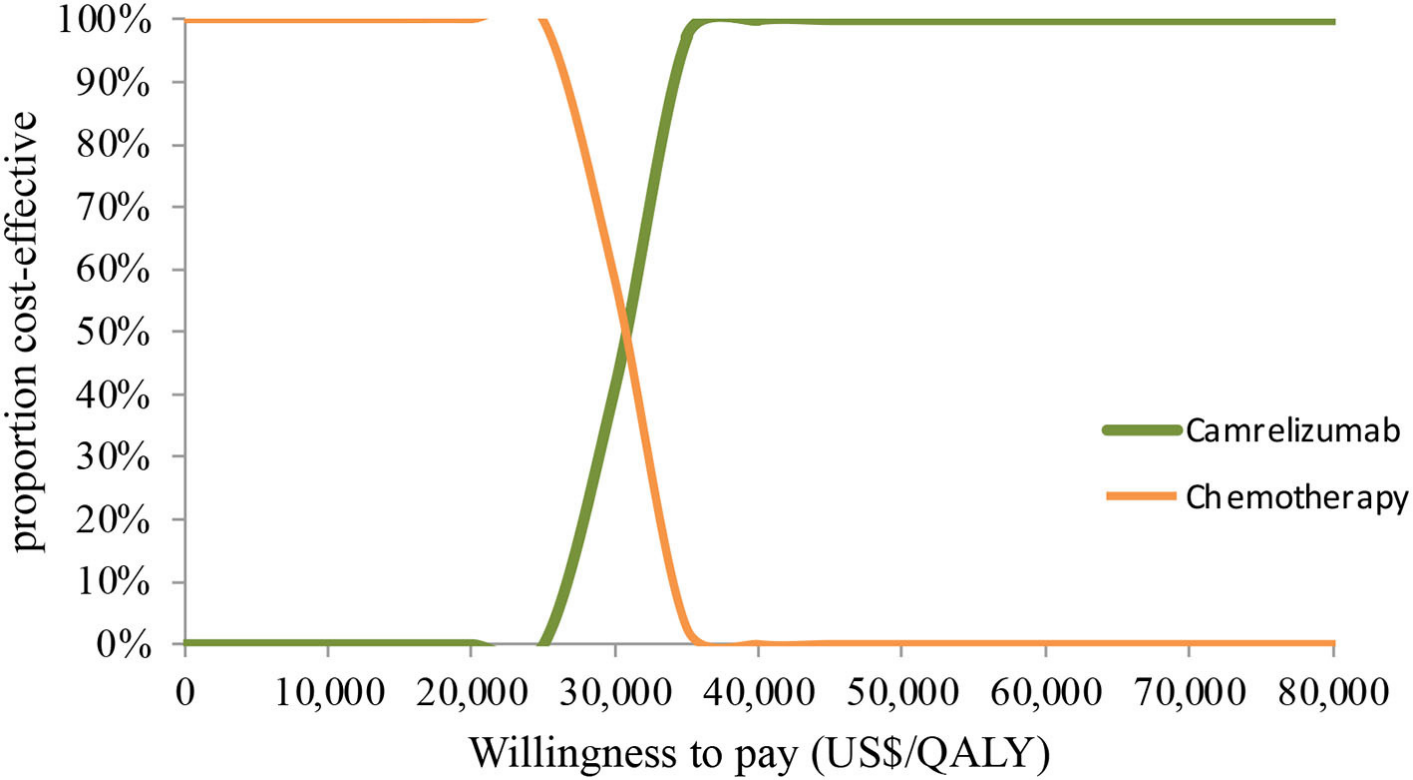
Cost-effectiveness acceptability curve for the scenario analysis. QALY, quality-adjusted life-years.

In PSA for both unselected patients and the patient subgroup with PD-L1 positive tumors, by Monte Carlo simulations, camrelizumab combination therapy was cost-effective in none of the 1,000 iterations, respectively (Figures 4, 5). This treatment regimen had a 50% probability of being cost-effective at a WTP threshold of around $63,000 per QALY and $48,140 per QALY in the base case and subgroup analyses, respectively. In the scenario, camrelizumab combination therapy would be cost-effective at a chance of 62.8% (Figure 6). If the WTP threshold increased to 39,000 per QALY, the probability of camrelizumab combination therapy to be cost-effective increased to 100%.

## Discussion

Camrelizumab combination therapy was approved as first-line treatment for advanced NSCLC by the NMPA of China in 2020 (9). As the first domestic PD-1 antibody for lung cancer, its approval represents a landmark significance for Chinese patients suffering from NSCLC. More encouragingly, in order to be included in NRDL, the price of camrelizumab has fallen significantly after price negotiation. However, whether the therapeutic schedule is cost-effective has not been confirmed. This study aims to fill this gap and provides an evidence-based assessment for the cost-effectiveness of camrelizumab combination therapy as first-line treatment of advanced non-squamous NSCLC in China.

Based on our model, compared to chemotherapy at a WTP threshold of $31,500 per QALY, camrelizumab combination therapy was not cost-effective for patients unselected by PD-L1 tumor expression with an ICER of $63,080 per QALY. The most influential factors of the ICER were the cost of pemetrexed, the cost of camrelizumab, and the risk of neutrophil count decreased. Results from PSA showed a zero probability of ICER lower than the given threshold. The subgroup analysis revealed that, in patients with PD-L1-expressing tumors, camrelizumab combination therapy failed to cross the threshold of WTP again despite a more favorable cost-effectiveness associated with an increased incremental QALY compared to the base case. Due to ethical issues, the base case analysis included a high proportion of patients switching from chemotherapy to camrelizumab after progression in the CameL trial, in the context of the current reality that not yet approving second-line camrelizumab in China for advanced non-squamous NSCLC (23, 24). The crossover diluted the survival benefits associated with first-line camrelizumab (23, 24). The scenario analysis showed that camrelizumab combination therapy exhibited an ICER of $30,591 per QALY, which crossed the cost-effectiveness threshold.

As there is no general agreement on a cost-effectiveness ratio threshold for China, the present study adopted the WHO criteria of three times GDP per capita per QALY ($31,500 per QALY) (25). Prior to camrelizumab, previous studies reported that immunotherapies with innovative PD-1/PD-L1 antibodies failed to show cost-effectiveness of $93,307 per QALY for nivolumab (13), $96,644 per QALY for pembrolizumab (26), and $325,329 per QALY for atezolizumab (12) in the first-line setting, suggesting their costs poorly rendered corresponding clinical value in China. Compared to these overpriced PD-1/PD-L1 antibodies, our findings demonstrated that the price reduction of camrelizumab provides a more promising opportunity to balance the efficacy and costs as first-line treatment against advanced NSCLC in resource-limited countries such as China.

The high cost of immunotherapies in anti-cancer treatment has been a long-term issue globally, also and particularly in low- and middle-income countries (27). To bring down prices and thus improved affordability of medicines, the Chinese government has overhauled its policy toolbox by promoting universal health insurance (28), expanding investment on biosimilar medicines (29), and conducting medical centralized volume procurement (30). The undergoing reforms has established a unified purchasing system, which significantly reversed the fragmentation in the health care system caused by hospitals individually purchasing. The super payer, China's National Health Security Administration (NHSA), takes advantage of the bargaining power in negotiating with pharmaceutical companies, resulting in an average price cut of more than 50% across 157 drugs (31–33). For example, to receive a definite volume commitment during the contract period (50–80% of total Chinese market), the domestic producer of camrelizumab reduce its price from a yearly cost of $53,191–$7,842 (34). This price negotiation, to a great extent, improved the affordability and accessibility of immunotherapies in China. Apart from camrelizumab, tislelizumab, one of the four domestically made PD-1 antibodies reported an 80% discount for landing on China's reimbursement list in 2020, had been approved for advanced non-squamous NSCLC recently. At the list price in China, those domestically made PD-1/PD-L1 antibodies would likely to have a very high chance to be cost-effective from perspectives of high-income countries such as the US and some members of the European Union.

In addition, the present study found that a high level of within-trial crossover underestimated the cost-effectiveness of this new regimen. The high proportion of within-trial crossover prolonged the median OS of chemotherapy, with 20.5 months compared to 15.2 months if OS data were adjusted by the RPSFT model (7, 8). Following adjustment for crossover, the OS HR associated with camrelizumab combination therapy compared to chemotherapy alone was 0.56 compared to 0.73 when unadjusted, implying that an analysis using the adjusted survival data would accord a substantial clinical value to camrelizumab. Following the improved clinical value, the adjusted OS contributed to higher estimates for QALYs gained associated with camrelizumab combination therapy than chemotherapy alone. The switching effect diluted the clinical value of camrelizumab and increased the cost of the chemotherapy group. Compared to the base case analysis, the crossover adjustment was associated with a larger magnitude of the incremental increase in QALYs than that of the incremental decrease in costs, contributing to the ICER dropped below the given WTP threshold.

In retrospect, the present study has several limitations. First, the present study derived the quality of life data from published literatures since those were unavailable in the CameL trial. Second, the costs were estimated inaccurately on account of ignoring indirect costs and management costs. The model only calculated the direct costs including the cost for drugs, management of AEs, routine follow-up, best supportive care, and palliative care. Third, data were unavailable for modeling health state or treatment-specific non-drug disease management costs and these costs may have been over-estimated for camrelizumab combination therapy relative to chemotherapy. Forth, the distribution of patients receiving post-progression therapy was simulated by the CameL trial. However, given that camrelizumab was approved by NMPA as first-line treatment, but not second-line treatment for NSCLC patients, a high proportion of crossover to camrelizumab monotherapy may not reflect the real-world situation, where patients have to receive other more expensive PD-1/PD-L1 antibodies after progression (35). Finally, the present study missed an analysis adjusting the crossover effect in patients with PD-L1 tumor expression, despite the fact that selecting PD-L1 tumor expression and the bias removal of within-trial crossover would synergistically move ICERs toward the given threshold. However, as the Camel trial has not reported the crossover-adjusted OS of PD-L1 positive patients, the present study could not conduct the additional scenario analysis in such patients.

## Conclusion

From the perspective of China's healthcare system, the camrelizumab combination therapy, regardless of the selection for PD-L1 tumor expression, was not cost-effective in comparison to chemotherapy as first-line treatment for NSCLC patients. However, upon crossover adjustment, camrelizumab combination therapy dropped below the WTP threshold and would be cost-effective for unselected patients at a chance of 62.8%. Future real-world study and cost-effectiveness analysis are warranted in PD-L1 positive patients with crossover adjustment, in which this new regimen would have a higher chance to become cost-saving.

## Data Availability Statement

The original contributions presented in the study are included in the article/Supplementary Material, further inquiries can be directed to the corresponding authors.

## Author Contributions

The conception and design of the study were primarily conducted by YZ and FC. The drafting of the paper was mainly the responsibility of GX and LG. All authors have reviewed the analysis and interpretation of the data and contributed to the drafting of the manuscript, revising the manuscript for important intellectual content, approved the final version to be published, and agree to be accountable for all aspects of the work.

## Funding

This study was supported by the National Natural Science Foundation of China under Grant 72004231, Science and Technology Plan of Nantong under Grant MS22020019, and the Multicenter Clinical Studies Program of Affiliated Hospital of Nantong University under Grant LCYJ-A10.

## Conflict of Interest

The authors declare that the research was conducted in the absence of any commercial or financial relationships that could be construed as a potential conflict of interest.

## Publisher's Note

All claims expressed in this article are solely those of the authors and do not necessarily represent those of their affiliated organizations, or those of the publisher, the editors and the reviewers. Any product that may be evaluated in this article, or claim that may be made by its manufacturer, is not guaranteed or endorsed by the publisher.
