# Supplementary material for: Economic Evaluation of First-Line Camrelizumab for Advanced Non-small-cell Lung Cancer in China
Source: Front Public Health. 2021 Dec 10;9:743558. doi: 10.3389/fpubh.2021.743558 (PMC8702426; doi:10.3389/fpubh.2021.743558)
Supplement: Supplementary file 1 [file Data_Sheet_1.ZIP › Table 1.docx]

Table 1 Model Inputs

| **Parameter** | |  | **Base case** | | | **Range** | | | | | | | | **Distribution** | | | | | | | **Source** | | |  |  |  |  |  |  |  |  |  |  |
| --- | --- | --- | --- | --- | --- | --- | --- | --- | --- | --- | --- | --- | --- | --- | --- | --- | --- | --- | --- | --- | --- | --- | --- | --- | --- | --- | --- | --- | --- | --- | --- | --- | --- |
|  | |  |  |  |  | **Low** | | | | **High** | | | |  |  |  |  |  |  |  |  |  |  |  |  |  |  |  |  |  |  |  |  |
| Treatment cost ($) | | | | | | | |  | | | |  | | | |  | |  | | | | | | |  | | | | | | | |  |
|  | Camrelizumab per cycle | | | 452.08 | | | 361.66 | | | | 542.50 | | | | Gamma | | | Local market | | | | | | | | | | | | |  |  |  |
|  | Carboplatin per cycle | | | 17.65 | | | 14.12 | | | | 21.18 | | | | Gamma | | | Local market | | | | | | | | | | | | |  |  |  |
|  | Pemetrexed per cycle | | | 1103.30 | | | 882.64 | | | | 1323.96 | | | | Gamma | | | Local market | | | | | | | | | | | | |  |  |  |
|  | Docetaxel per cycle | | | 94.10 | | | 75.28 | | | | 112.92 | | | | Gamma | | | Local market | | | | | | | | | | | | |  |  |  |
|  | Gefitinib per cycle | | | 161.47 | | | 129.18 | | | | 193.76 | | | | Gamma | | | Local market | | | | | | | | | | | | |  |  |  |
|  | Bevacizumab per cycle | | | 1788.42 | | | 1430.73 | | | | 2146.10 | | | | Gamma | | | Local market | | | | | | | | | | | | |  |  |  |
|  | Nivolumab per cycle | | | 4283.44 | | | 3426.75 | | | | 5140.13 | | | | Gamma | | | Local market | | | | | | | | | | | | |  |  |  |
|  | Supportive care per cycle | | | 338.00 | | | 270.40 | | | | 405.60 | | | | Gamma | | | (15) | | | | | | | | | | | | |  |  |  |
|  | Routine follow-up per cycle^a^ | | | 85.71 | | | 68.57 | | | | 102.85 | | | | Gamma | | | (15) | | | | | | | | | | | | |  |  |  |
|  | Palliative care per event | | | 2464.50 | | | 1971.60 | | | | 2957.40 | | | | Gamma | | | (15) | | | | | | | | | | | | |  |  |  |
| Cost of managing adverse events ($) | | | | |  | | | |  | | | |  | | | |  | | | | |  | | | | | | | | | | |  |
|  | Neutrophil count decreased | | | 175.37 | | | 140.30 | | | | 210.44 | | | | Gamma | | | (16) | | | | | | | | | |  |  |  |  |  |  |
|  | Anemia | | | 101.02 | | | 80.82 | | | | 121.22 | | | | Gamma | | | (17) | | | | | | | | | |  |  |  |  |  |  |
|  | Platelet count decreased | | | 603.79 | | | 483.03 | | | | 724.55 | | | | Gamma | | | (18) | | | | | | | | | |  |  |  |  |  |  |
| Risk of adverse events in  camrelizumab group (grade III–IV) | | | | | | | |  | | | |  | | | |  | |  | | | | | | | | | | | | | |  | |
|  | Neutrophil count decreased | | | 0.38 | | | 0.34 | | | | 0.42 | | | | Beta | | | | | (7) | | | | | | | | | |  |  |  |  |
|  | Anemia | | | 0.19 | | | 0.17 | | | | 0.21 | | | | Beta | | | | | (7) | | | | | | | | | |  |  |  |  |
|  | Platelet count decreased | | | 0.17 | | | 0.15 | | | | 0.19 | | | | Beta | | | | | (7) | | | | | | | | | |  |  |  |  |
| Risk of adverse events in  chemotherapy group (grade III–IV) | | | | | | | |  | | | |  | | | |  | |  | | | | | | | | | | | | | |  | |
|  | Neutrophil count decreased | | | 0.30 | | | 0.27 | | | | 0.33 | | | | Beta | | | | (7) | | | | | | | | | |  |  |  |  |  |
|  | Anemia | | | 0.11 | | | 0.10 | | | | 0.12 | | | | Beta | | | | (7) | | | | | | | | | |  |  |  |  |  |
|  | Platelet count decreased | | | 0.12 | | | 0.11 | | | | 0.13 | | | | Beta | | | | (7) | | | | | | | | | |  |  |  |  |  |
| Health utility | | | | | | | |  | | | |  | | | |  | |  | | | | | | | | | | | | | |  | |
|  | Stable disease | | | 0.81 | | | 0.73 | | | | 0.90 | | | | Beta | | | (19) | | | | |  |  |  |  |  |  |  |  |  |  |  |
|  | Disease progression | | | 0.58 | | | 0.52 | | | | 0.64 | | | | Beta | | | (20) | | | | |  |  |  |  |  |  |  |  |  |  |  |
| Health disutility | | | | | | | |  | | | |  | | | |  | |  | | | | | | | | | | | | | |  | |
|  | Neutrophil count decreased | | | 0.20 | | | 0.18 | | | | 0.22 | | | | Beta | | | (21) | | | | | | | |  |  |  |  |  |  |  |  |
|  | Anemia | | | 0.07 | | | 0.07 | | | | 0.08 | | | | Beta | | | (21) | | | | | | | |  |  |  |  |  |  |  |  |
|  | Platelet count decreased | | | 0.11 | | | 0.10 | | | | 0.12 | | | | Beta | | | (22) | | | | | | | |  |  |  |  |  |  |  |  |
|  | Discount rate | | | 0.05 | | | 0.00 | | | | 0.08 | | | | Fixed in PSA | | | — | | | | | |  |  |  |  |  |  |  |  |  |  |
|  | | | | | | | | | | | | | | | | | |  | | | | | |  | | |  |  |  |  |  |  |  |

Abbreviations: PSA, probabilistic sensitivity analysis.
