# Supplementary material for: Economic Evaluation of First-Line Camrelizumab for Advanced Non-small-cell Lung Cancer in China
Source: Front Public Health. 2021 Dec 10;9:743558. doi: 10.3389/fpubh.2021.743558 (PMC8702426; doi:10.3389/fpubh.2021.743558)
Supplement: Supplementary file 1 [file Data_Sheet_1.ZIP › Table 2.docx]

Table 2 Discounted Incremental Cost-Effectiveness of Camrelizumab

|  |  |  |  |  | | Incremental | | | | | | ICER (incremental cost/QALY, $) |
| --- | --- | --- | --- | --- | --- | --- | --- | --- | --- | --- | --- | --- |
| Analysis | | Total cost, $ | LYs | QALYs | | Cost, $ | | LYs | | QALYs | |  |
| Base case | |  |  |  |  | |  | |  | |  | |
|  | Camrelizumab | 19,921 | 1.36 | 0.86 | 6,938 | | 0.18 | | 0.11 | | 63,080 | |
|  | Chemotherapy | 12,983 | 1.18 | 0.75 | NA | | NA | | NA | | NA | |
| Sensitivity analysis | |  |  |  |  | |  | |  | |  | |
|  | Camrelizumab | 20,631 | 1.54 | 0.99 | 5,738 | | 0.18 | | 0.12 | | 46,311 | |
|  | Chemotherapy | 14,894 | 1.36 | 0.88 | NA | | NA | | NA | | NA | |
| Scenario analysis | |  |  |  |  | |  | |  | |  | |
|  | Camrelizumab | 19,921 | 1.36 | 0.86 | 10,508 | | 0.55 | | 0.34 | | 30,591 | |
|  | Chemotherapy | 9,413 | 0.81 | 0.52 | NA | | NA | | NA | | NA | |

Abbreviations: LYs, life-years; QALYs, quality-adjusted life-years; ICER, incremental cost-effectiveness ratio; NA, not applicable.
