# Supplementary material for: Economic Evaluation of First-Line Camrelizumab for Advanced Non-small-cell Lung Cancer in China
Source: Front Public Health. 2021 Dec 10;9:743558. doi: 10.3389/fpubh.2021.743558 (PMC8702426; doi:10.3389/fpubh.2021.743558)
Supplement: Supplementary file 1 [file Data_Sheet_1.ZIP › Supplemental materials.docx]

Economic Evaluation of First-Line Camrelizumab for Advanced Non-Small-Cell Lung Cancer in China

Table S1 Summary of AIC and BIC statistics for modeling OS curve of patient unselected by PD-L1 tumor expression

| Fitted Function | Camrelizumab | | Chemotherapy without switching | | Chemotherapy with switching adjustment adjustment | |
| --- | --- | --- | --- | --- | --- | --- |
|  | AIC | BIC | AIC | BIC | AIC | BIC |
| Exponential | 856.2032 | 859.4813 | 981.7069 | 985.0445 | 952.1914 | 955.5289 |
| Weibull | 842.5556 | 849.1118 | 975.9464 | 982.6215 | **910.658** | **917.3331** |
| Log normal | **837.1728** | **843.729** | **973.3517** | **980.0267** | 934.7852 | 941.4603 |
| log-logistic | 881.9132 | 888.4694 | 1034.483 | 1041.158 | 930.7153 | 937.3904 |

Table S2 Summary of AIC and BIC statistics for modeling PFS curve of patient unselected by PD-L1 tumor expression

| Fitted Function | Camrelizumab | | Chemotherapy without switching adjustment | |
| --- | --- | --- | --- | --- |
|  | AIC | BIC | AIC | BIC |
| Exponential | 834.9621 | 838.2402 | 928.2171 | 931.5689 |
| Weibull | 827.3263 | 833.8826 | 927.2503 | 933.9541 |
| Log normal | **826.8288** | **833.385** | **915.9247** | **922.6284** |
| log-logistic | 873.241 | 879.7973 | 1010.331 | 1017.035 |

Table S3 Summary of AIC and BIC statistics for modeling OS curve of patient with PD-L1-expressing tumors (≥1%)

| Fitted Function | Camrelizumab | | Chemotherapy without switching adjustment | |
| --- | --- | --- | --- | --- |
|  | AIC | BIC | AIC | BIC |
| Exponential | 510.0049 | 512.8647 | 493.1331 | 495.8426 |
| Weibull | 509.8689 | 515.5885 | 494.589 | 500.008 |
| Log normal | **508.0381** | **513.7577** | **492.5209** | **497.9399** |
| log-logistic | 540.1503 | 545.8699 | 530.9715 | 536.3906 |

Table S4 Summary of AIC and BIC statistics for modeling PFS curve of patient with PD-L1-expressing tumors (≥1%)

| Fitted Function | Camrelizumab | | Chemotherapy without switching adjustment | |
| --- | --- | --- | --- | --- |
|  | AIC | BIC | AIC | BIC |
| Exponential | 497.3492 | 500.2012 | 496.6232 | 499.3939 |
| Weibull | **493.0904** | **498.7945** | 498.5471 | 504.0884 |
| Log normal | 494.2873 | 499.9913 | **493.1257** | **498.667** |
| log-logistic | 517.4119 | 523.1159 | 549.9088 | 555.4501 |

Table S5 Distribution of post-discontinuation therapy in the model

| Post-discontinuation regimen | Camrelizumab | Chemotherapy without adjustment | Chemotherapy with switching adjustment |
| --- | --- | --- | --- |
| Gefitinib | 0.3 |  |  |
| Docetaxel | 0.55 | 0.28 | 1.00 |
| Docetaxel+bevacizumab | 0.15 |  |  |
| Camrelizumab |  | 0.66 |  |
| Nivolumab |  | 0.06 |  |
